# Brain Complexity and Parametrization of Power Spectral Density in Children with Specific Language Impairment

**DOI:** 10.3390/e27060572

**Published:** 2025-05-28

**Authors:** Brenda Y. Angulo-Ruiz, Elena I. Rodríguez-Martínez, Francisco J. Ruiz-Martínez, Ana Gómez-Treviño, Vanesa Muñoz, Sheyla Andalia Crespo, Carlos M. Gómez

**Affiliations:** 1 Human Psychobiology Laboratory, Experimental Psychology Department, University of Seville, 41018 Seville, Spain; elisroma@us.es (E.I.R.-M.); frmartinez@us.es (F.J.R.-M.); lmunnoz@us.es (V.M.); sheandcre@alum.us.es (S.A.C.); cgomez@us.es (C.M.G.); 2 Unidad de Desarrollo Infantil y Atención Temprana (UDIATE) Affiliated with the Hospital Victoria Eugenia, Spanish Red Cross, 41009 Seville, Spain; ana.gomezt@cruzroja.es

**Keywords:** multiscale entropy, parameterized PSD, aperiodic component, periodic component, specific language impairment

## Abstract

This study examined spontaneous activity in children aged 3–11 years with specific language impairment (SLI) using an electroencephalogram (EEG). We compared SLI-diagnosed children with a normo-development group (ND). The signal complexity, multiscale entropy (MSE) and parameterized power spectral density (FOOOF) were analyzed, decomposing the PSD into its aperiodic (AP, proportional to 1/fx) and periodic (P) components. The results showed increases in complexity across scales in both groups. Although the topographic distributions were similar, children with SLI exhibited an increased AP component over a broad frequency range (13–45 Hz) in the medial regions. The P component showed differences in brain activity according to the frequency and region. At 9–12 Hz, ND presented greater central–anterior activity, whereas, in SLI, this was seen for posterior–central. At 33–36 Hz, anterior activity was greater in SLI than in ND. At 37–45 Hz, SLI showed greater activity than ND, with a specific increase in the left, medial and right regions at 41–45 Hz. These findings suggest alterations in the excitatory–inhibitory balance and impaired intra- and interhemispheric connectivity, indicating difficulties in neuronal modulation possibly associated with the cognitive and linguistic characteristics of SLI.

## 1. Introduction

Specific language impairment (SLI) is classified within Communication Disorders in the DSM-V. It incorporates expressive language disorder and mixed receptive and expressive language disorder from the DSM-IV edition. Within this classification, SLI would be embedded in the Developmental Language Disorder category in the DSM-V [1,2]. It is defined as an impairment in language acquisition and use, arising from inappropriate social conditions, in the absence of physical, cognitive, sensory or social–affective deprivation [3]. Research suggests that these difficulties are influenced by genetic factors [4,5,6,7]. Diagnosis is most common in childhood, with an international prevalence ranging from 1.4% to 16.2% of the school population [3,8]. In Andalusia (Spain), the prevalence ranges from 5.26% to 12.58% [3]. SLI often leads to reading difficulties, such as dyslexia [9], and negatively impacts academic performance [10]. Furthermore, it has been associated with emotional, behavioral and social difficulties during adolescence [11,12,13], as well as a significant reduction in quality of life and well-being in adulthood [14].

Several models have been proposed to understand the underlying mechanisms of SLI. Among these, the speed-of-processing limitation hypothesis [15] suggests that children with SLI have difficulty processing rapid auditory stimuli, which impairs their ability to discriminate phonemes with brief transitions and consequently affects language development. Meanwhile, the statistical learning model [16] proposes that individuals with SLI have difficulty extracting implicit regularities from sequences of stimuli, which may explain their difficulties in acquiring grammatical rules. Finally, the temporal processing hypothesis of language [17] proposes that disruptions in neural synchronization with the rhythmic structures of language may serve as a neurobiological basis for SLI. In other words, difficulties in aligning neural activity with speech rhythms may impair the perception of prosodic and phonological patterns, contributing to deficits in language development. Taken together, these models suggest that SLI possibly is not just a language disorder but also involves impairments in learning and auditory processing.

Neurobiological studies in SLI have shown structural, functional and connectivity changes in key brain regions involved in language processing [18]. At a global level, the findings are inconsistent: while some studies report a reduced total brain volume in SLI [19], others find an increased volume [20]. This discrepancy may be explained by variations in cortical morphology, suggesting that the primary affected variable is the surface area rather than the cortical thickness, with lower values observed in the SLI group [21]. At a regional level, an increase in the volume of the left inferior frontal cortex and a decrease in the right caudate nucleus and superior temporal cortex has been observed in children with SLI [22]. Furthermore, in children under 11 years of age, more extensive growth patterns were identified in regions such as the entorhinal, temporopolar, motor-precentral and precuneus cortex [23], as well as structural enlargement in subcortical regions such as the putamen, nucleus accumbens and right globus pallidus [24]. At the microstructural level, based on diffusion tensor imaging (DTI), an increase in white matter in the frontal and temporal regions has been reported [23], as well as reductions in fractional anisotropy (FA) in the globus pallidus and thalamus [24]. In addition, it has been shown that the dorsal striatum has reduced myelin content, which may reduce the neuronal transmission efficiency [25]. In terms of connectivity, findings suggest an abnormal increase in white matter in children with SLI, indicating impaired connectivity across neural networks [18,20,26].

From a functional perspective, atypical patterns of lateralization and activation have been described in the left hemisphere, with reduced or absent activation in the left inferior frontal gyrus and compensatory activation in the right, according to functional magnetic resonance imaging (fMRI) studies [27,28]. In addition, research using single-photon emission computed tomography (SPECT) has shown the abnormal lateralization of blood flow patterns and/or hypoperfusion in language-related regions [29], while studies using functional transcranial Doppler (fTCD) have shown the right hemisphere lateralization of language or a bilateral distribution in SLI [30]. These studies have linked specific language deficits to the atypical development of both ventral [31] and dorsal [32,33] language streams. Furthermore, a persistent deficit in the maturation of these pathways has been proposed in SLI [33].

Neurophysiological studies using electroencephalograms (EEGs) and computing event-related potentials (ERPs) have identified deficits in children with SLI [34]. These studies highlight limitations in the attentional discrimination of auditory stimuli, evidenced by a reduced or absent mismatch negativity (MMN) component [35,36,37]. Specific deficits have been observed in lexical access and semantic integration, with a delayed [35] or absent N400 component [38,39]. Impaired speech processing and differences in the allocation of attentional resources to auditory stimuli have been reported, reflected in a delayed P100 component [40]. Difficulties in central auditory processing have also been noted, as indicated by alterations in the T-complex [41], which is a bilateral negative component at temporal sites [42], and the P300 component [43]. Furthermore, deficits in processing and storing visual information in working memory have been evidenced by a decreased P3b amplitude [44].

Resting-state EEG studies have analyzed the signal using both linear (power spectral density [PSD]) and non-linear (multiscale entropy [MSE]) measures in ND subjects and those with neurodevelopmental disorders. Regarding the PSD, during typical brain maturation, the absolute spectral power decreases, while the relative spectral power fluctuates, reflecting a transition from slow (delta and theta) to fast (alpha, beta, and gamma) frequencies. This shift is associated with synaptic pruning, which optimizes neuronal transmission [45,46,47,48,49,50]. As for MSE, maturation is characterized by increased values at fine scales and decreased values at coarse scales, indicating the strengthening of local connections and the refinement of long-range connections with age [51,52].

In neurodevelopmental disorders such as attention-deficit hyperactivity disorder (ADHD) and autism spectrum disorder (ASD), the typical maturation pattern is altered. In ADHD, the PSD increases in specific frequency bands, including delta and theta [50,53,54,55], and this occurs in theta, alpha and beta in ASD [56,57,58,59,60]. Regarding MSE, studies indicate a less consistent maturation pattern and lower values in both ADHD [61,62,63,64] and ASD [65,66,67,68]. However, the literature remains inconsistent for both PSD [53,69,70] and MSE [71,72]. For SLI, although research is more limited, studies report increased delta and theta band activity (relative PSD [73]; absolute PSD [74]; absolute PSD [75]) and reduced alpha and beta band PSD values (relative PSD [73]; absolute PSD [75]; absolute PSD [76]). However, recent findings suggest higher spectral power in the beta band in children with SLI compared to ND children (absolute PSD [75]). As far as we know, complexity measures have not been described for the analysis of resting-state EEG in SLI. This lack of evidence, combined with inconsistencies in the PSD literature, underscores the need for further research to clarify the underlying neurophysiological mechanisms of SLI.

Recently, an innovative approach to PSD analysis has been proposed, offering a more detailed and accurate neurophysiological assessment while minimizing information loss in the interpretation of cognitive and behavioral states [77,78]. This method involves decomposing the PSD into two components: the aperiodic component (AP), characterized by a 1/f-like distribution, which provides insights into the neuronal activation level (offset parameter) and the balance between excitatory and inhibitory synaptic currents (exponent parameter) [79,80], and the periodic component (P), which reflects genuine rhythmic patterns of brain activity [77]. This decomposition is based on the assumption that the EEG power spectrum contains oscillatory activity overlaid on a scale-free background signal. Traditional spectral analyses often combine these components, which can obscure physiologically relevant information. By modeling and subtracting the aperiodic component, the newly proposed method isolates the actual oscillatory peaks, allowing for the more accurate characterization of frequency-specific activity. Technically, this approach is commonly implemented using algorithms such as Fitting Oscillations and One Over F (FOOOF), which iteratively fits the aperiodic background and identifies periodic peaks that exceed the aperiodic estimate [77,78]. These components appear to be altered in neurodevelopmental disorders such as ADHD [81,82,83,84] and ASD [85].

The present study aimed to apply this new PSD decomposition methodology to a sample of children with SLI compared to ND children to examine more precisely the patterns of neural activity underlying this disorder. Additionally, given the lack of previous studies on brain signal complexity in SLI, MSE was explored to assess the stability and adaptability of neural activity. We expected to observe an increase in MSE with the order of the scale in both groups, with lower complexity values in the SLI group. Regarding the PSD components, we hypothesized that there would be group differences in the AP and P components, primarily in language-related regions and at the most relevant frequencies, similarly to those observed in canonical PSD analyses [73,75,76]. The selection of resting-state EEG responds to the need for a neurophysiological assessment that is not dependent on task-specific performance, which is especially important in populations with language difficulties, such as children with SLI. These analyses were computed in a broad age range (3–11 years) given the extension of the SLI impairment across ages and in order to obtain a sufficient number of SLI subjects.

## 2. Materials and Methods

### 2.1. Sample

A total of 66 subjects participated in this study, divided into two groups: a normo-development (ND) group and a clinical group diagnosed with specific language impairment (SLI). The SLI group consisted of 30 children aged 3 to 10 years (M = 6.37, SD = 1.75, 19 males). Participants were recruited from the Unidad de Desarrollo Infantil y Atención Temprana (UDIATE: https://hospitalveugenia.com/udiate-atencion-temprana-desarrollo-infantil/ accessed on 1 April 2025), affiliated with the Hospital Victoria Eugenia, part of the Spanish Red Cross, which specializes in the assessment and treatment of neurodevelopmental disorders.

The ND group consisted of a total of 36 participants aged 3 to 11 years (M = 6.69, SD = 2.08, 19 males). This group was recruited from different schools in Seville. Parents did not report any neurological diseases, signs of epileptic discharge, learning difficulties or developmental delays in the children.

The experimental protocol was approved by the biomedical research ethics committee of the Autonomous Community of Andalusia, following the guidelines of the Declaration of Helsinki (code: 0818-N-21). Written informed consent was obtained from the parents, who were provided with written information and an explanation regarding the objectives and characteristics of the study.

### 2.2. Psychological Test

To be included in the study, SLI children required a clinical report confirming their language deficits, supported by language experts from the clinical center (see above) based on tests such as the *Clinical Evaluation of Language Fundamentals-5* (*CELF-5*; [86]), the *Navarre Oral Language Test—Revised* (*PLON-R*; [87]), the *Illinois Test of Psycholinguistic Abilities* (*ITPA*; [88]), the *Peabody Picture Vocabulary Test* (*PPVT-5*; [89]) or the *Kaufman Brief Intelligence Test* (*KBIT*; [90]) or a structured interview following the *DSM-V* or *CIE-10* criteria, all of which were administered by language therapists. Previously, otorhinolaryngologists did not find basic auditory problems according to audiometry and/or brainstem auditory evoked potentials.

The *KBIT* [90] was used to assess the non-verbal cognitive skills of all children participating in the study.

### 2.3. EEG Recording

Spontaneous brain electrical activity was recorded using EEG for three minutes while participants had their eyes open. Participants were asked to sit in a comfortable position and look at an hourglass. This element was used as a passive aid for visual fixation, minimizing eye movements and maintaining a stable resting state. Given the young age of many participants, traditional fixation strategies, such as the cross on a blank screen, were not considered optimal for maintaining attention. The hourglass provided a slow and engaging visual stimulus that helped to maintain gaze stability, which promoted consistency in the recording conditions across participants. The recording was obtained from 19 electrodes mounted on an electrode cap (ELECTROCAP), selected from the international 10–20 system (Fp1, Fp2, F3, F4, C3, C4, P3, P4, O1, O2, F7, F8, T3, T4, T5, T6, Cz, Fz, Pz). For all participants, an average reference was used, and impedance was kept below 10 kΩ. Data were recorded in direct current at 1024 Hz, without any filtering. The amplification gain was set to 20,000 using an analog-to-digital acquisition and analysis system (ANT Amplifiers, The Netherlands).

### 2.4. Data Analysis

#### 2.4.1. EEG Pre-Processing

The EEGLAB software package [91] and Matlab R2021b were used to analyze the raw EEG data. Pre-processing consisted of applying (i) a 47–53 Hz notch filter (EEGLAB function: *eegfiltnew*), (ii) an average reference and (iii) an artifact subspace reconstruction (*ASR*) algorithm (EEGLAB function: *clean_raw_data*). ASR was used to correct segments of the data with a standard deviation greater than 20 times that of the calibration data [92]. The epochs had a duration of 4 s (4000 ms). Eyeblink, muscle and other movement artifacts were removed using independent component analysis (*ICA*; function: *pop_runica*) with the natural gradient function [93,94] and the ICLabel extension classification [95] in EEGLAB. The EEG signal was reconstructed, and all epochs with amplitudes exceeding ±120 μV were rejected using the *eegthresh* function.

#### 2.4.2. Multiscale Entropy

Multiscale entropy (MSE) was calculated for all EEG participants and electrodes using the *Multiscale Sample Entropy* function in MATLAB [96], based on the MSE method proposed by Costa et al. [97]. This method measures the signal complexity by calculating the sample entropy (SE; [98]) on multiple time scales using a coarse-graining procedure. MSE provides an estimate of complexity by dividing the EEG signal into non-overlapping windows of different sample sizes and evaluating the repetition frequency of patterns of length ‘m’ compared to patterns of length ‘m + 1’. To calculate MSE, each time scale is defined by averaging neighboring points (p) in the original time series (length τ). A similarity limit (r) is set to define the tolerance range within which neighboring points are considered similar (k), and this limit is normalized to the standard deviation (SD) of the EEG, according to the relationship k < r × SD [96]. Sample entropy is calculated for each time scale according to formula (1):

(1) $$SE=\log\frac{p^{m}\left(r\right)}{p^{\left(m+1\right)}\left(r\right)}$$

Following previous recommendations in studies on EEG signal complexity [98,99,100,101,102], the parameters were set to m = 2 and r = 0.5. The MSE was calculated for 136 time scales, corresponding to 30 point windows obtained by collapsing 136 consecutive points sampled in each 4-s trial (0.97656 ms × 136 scales = 132.81 ms). A detailed description of the number of points, sample periods and frequencies covered in MSE is provided in Supplementary Table S1. High MSE values are associated with high complexity, indicating less repetition and greater diversity of patterns at different time scales, suggesting an information-rich signal [103]. In contrast, low MSE values reflect greater regularity or predictability in the signal patterns, which may indicate a reduction in information richness [99,104].

In the MSE calculation, the coarse-graining procedure acts as a filter for higher frequencies as the time scales increase. This implies that, at lower scales, all frequencies present in the EEG signal are included, while, at higher scales, only low frequencies are retained. Recently, this approach has been linked to power spectrum analysis using methods such as Haar wavelets, which also separate frequencies into different levels [105].

#### 2.4.3. Parameterization of *Fitting Oscillations and One over F* (*FOOOF*)

A MATLAB-adapted version of the *Fitting Oscillations and One Over F* (*FOOOF*) function (version 3.8), originally implemented in Python, was used to analyze the power spectral density (PSD) data. The FOOOF *specparam* tool (https://fooof-tools.github.io/fooof/reference.html) [77] allowed us to decompose the spectrum (canonical PSD) into the full model fit (adjusted PSD), the aperiodic component (AP) and the periodic component (P). Given the high similarity between the canonical PSD and the fitted PSD observed in a previous study [106] and confirmed in the present data (Supplementary Figure S1), the present study focused exclusively on the adjusted PSD and the AP and P component analyses. The PSD is provided directly from *specparam*, which implements Welch’s method, incorporated into the EEGLAB *spectopo* function, to calculate the power spectral density in the range of 1 to 45 Hz.

The *FOOOF* algorithm was configured using the following recommended parameters: peak_width_limits = [1,8], min_peak_height = 0.05, peak_threshold = 0.5, max_n_peaks = 6 and aperiodic_mode = ‘fixed’ [77,78]. In this approach, the AP component computed from the offset and exponent parameters models the background activity in log–log space, while the P component represents the oscillations that stand out from this activity, modeled by Gaussian functions with peak power, central frequency and bandwidth parameters (see [77] for details). As *FOOOF* does not directly provide a fitted spectrum for the P component, it was obtained by subtracting the AP component (ap_fit in *specparam*) from the adjusted PSD (2):

(2) $$P=PSD\;adjusted-ap_{fit}$$

To assess the goodness-of-fit of the model across all subjects, the explained variance (R^2^) and mean absolute error (MAE) metrics were calculated, comparing the fitted model with the original power spectrum. The results showed the good fit of the algorithm in each data set, as shown in Supplementary Figure S1, following the fit criteria reported by Ostlund et al. [107] (MAE underfit > 0.1, MAE overfit < 0.020).

### 2.5. Statistical Analysis

The assumptions of normality and homogeneity of variances were tested using the Shapiro–Wilk and Levene tests, respectively. When these assumptions were violated, non-parametric analyses were conducted using the Mann–Whitney U test. This approach was applied to assess group differences in demographic and preprocessing variables such as age, biological sex, the number of remaining ICA components and epochs. For variables that met the parametric assumptions, such as the *KBIT* raw scores, independent-sample *t*-tests were used.

Statistical analyses were performed in SPSS v25 and MATLAB for multiscale entropy (MSE) and power spectrum component (PSD) parametrization using the Parametrization of *Fitting Oscillations and One Over F* (*FOOOF*) model. For both measurements, the analytical strategy for electrodes was a reduction in dimensionality by creating nine regions covering the whole scalp: left anterior, left central, left posterior, medial anterior, medial central, medial posterior, medial posterior, right anterior, right central and right posterior (Supplementary Table S2).

#### 2.5.1. Multiscale Entropy

The MSE was divided into three types of scales [52] in order to reduce the 136 scales to a lower resolution and dimensionality: (i) fine scales, ranging from scale 1 (0.976 ms, 4095 time points) to scale 25 (24.41 ms, 163 time points); (ii) medium scales, ranging from scale 26 (25.39 ms, 157 time points) to scale 46 (44.92 ms, 89 time points); and (iii) coarse scales, ranging from scale 47 (45.89 ms, 87 time points) to scale 136 (132.81 ms, 30 time points) in each group (ND and SLI). For the parameters of the different scales, see Supplementary Table S1. This classification reflects the underlying neural dynamics: fine scales capture high-frequency activity linked to local processing and tend to increase with age; coarse scales capture low-frequency activity related to long-range communication and tend to decrease with age; and medium scales reflect intermediate dynamics with mixed frequency contributions and typically show minimal age-related changes [52].

A repeated-measures analysis of variance (RM-ANOVA) was applied. Within-subjects factors were (i) scale (fine, medium and coarse); (ii) laterality (left, medial, right); and (iii) anteroposterior orientation (anterior, central, posterior). The intersubject factor was group (ND and SLI), with age in days and biological sex as covariates. The statistical power was also computed, and the effect size was calculated directly using the partial eta-squared (ηp^2^). Post hoc analyses were performed using Student’s *t*-test for pairwise comparisons, with Cohen’s d as the effect size metric [108] and correction for multiple comparisons using the False Discovery Rate method (FDR; [109]). Only significant results will be discussed when including the group factor, and the FDR-corrected post hoc analyses were significant. Some laterality effects are reported for their possible relation to language lateralization.

#### 2.5.2. Parametrization of Fitting Oscillations and One over F (FOOOF)

The aperiodic component (AP), its offset and exponent parameters and the periodic component (P) were analyzed separately. This decision was based on the recommendations of Donoghue et al. [77], who proposed to model these components independently in order to obtain a more accurate interpretation of the underlying neural dynamics. Although both components are derived from the same decomposition model (*FOOOF*), they reflect distinct neurophysiological processes: the AP component has been linked to the balance between excitation and inhibition, while the P component captures specific oscillatory activity. A topographical analysis was performed for each experimental group (ND and SLI), as well as for the differences between both groups (ND-SLI), on the parameters of both components (AP and P). For the P component, topographic maps were generated for 11 frequency bands (4 Hz windows: 1–4 Hz to 41–45 Hz). It should be noted that the last frequency range included frequencies up to 45 Hz.

Subsequently, independent RM-ANOVAs were performed for the AP component parameters (offset and exponent), as well as for the AP and P components, in each of the 11 frequency bands indicated above. In all cases, the same within-subject factors (laterality and anterior–posterior distribution), the between-subject factor (group: ND vs. SLI) and the covariates (age and biological sex) were included. Post hoc analyses were performed following the same criteria as described for the MSE analysis, including correction for multiple comparisons using FDR and calculation of the effect size with Cohen’s d.

In response to possible concerns about the sensitivity to outliers, a complementary analysis was performed using a standard z-score method (z > 3) to identify and correct for outliers by replacing them with the mean of the respective variable. This procedure was applied to the periodic and aperiodic components separately.

## 3. Results

### 3.1. Demographic, Cognitive and Technical Results of Participants

The normality of the age data was assessed using the Shapiro–Wilk test (appropriate for samples under 50 participants). The ND group showed a normal distribution (*p* = 0.191), whereas the SLI group did not (*p* < 0.001). Homogeneity of variances was confirmed with Levene’s test (*p* = 0.947). No significant age differences were found between groups (Mann–Whitney U = 547.5, Z = 0.097, *p* = 0.923).

As biological sex presented only two categories, they were considered as dummy variables, and no significant statistical differences were found in the subject sample (U = 597, Z = 0.857, *p* = 0.391). Consequently, age and biological sex are not considered relevant factors for the discussion of the results.

The *KBIT* raw scores met the assumptions of normality (ND: *p* = 0.132; SLI: *p* = 0.840) and homogeneity of variances (*p* = 0.900). The statistical analysis revealed a significant difference between the ND and SLI groups in cognitive skills (t(55) = 2.22, *p* = 0.030, d = 0.059) (Table 1).

Subjects’ recordings in the range of 20–45 epochs and 11–13 remaining components were accepted for analysis. The Shapiro–Wilk test revealed that the number of epochs in both groups did not follow a normal distribution (ND: *p* < 0.001; SLI: *p* < 0.001), which was also observed for the components (ND: *p* < 0.001; SLI: *p* < 0.001). Levene’s test indicated that homogeneity of variances was not satisfied for the epochs (*p* < 0.001) or for the components (*p* < 0.001). The Mann–Whitney U-test revealed significant differences in epochs (U = 315, Z = -2.99, *p* = 0.003) and components (U = 702, Z = 2.86, *p* = 0.004) between the groups.

Table 1 shows the means and standard deviations of the specific language impairment (SLI) and normo-developmental (ND) groups.

### 3.2. Multiscale Entropy (MSE)

**Figure 1:**
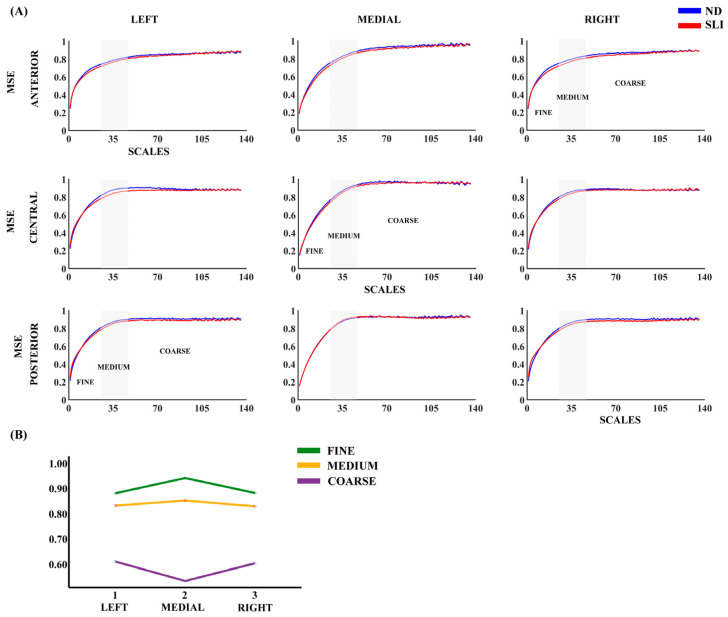
(**A**) Multiscale entropy (MSE) for nine considered areas in each group (normo-development (ND) and specific language impairment (SLI)). (**B**) Marginal means of MSE when collapsing the electrodes across the antero-posterior dimension to observe the laterality effects reported in the RM-ANOVA (Supplementary Table S3).

Figure 1 presents the results regarding multiscale entropy (MSE) across the nine evaluated areas, comparing participants from the ND and SLI groups. In both groups, we observed a consistent increase in MSE with the order of the scales across all considered areas. The RM-ANOVA analysis did not reveal significant effects between the groups. Some laterality effects are reported in the description of Supplementary Table S3 considering its possible relationship with language lateralization.

### 3.3. Parametrization of Fitting Oscillations and One over F (FOOOF)

#### 3.3.1. Aperiodic Component (AP)

**Figure 2:**
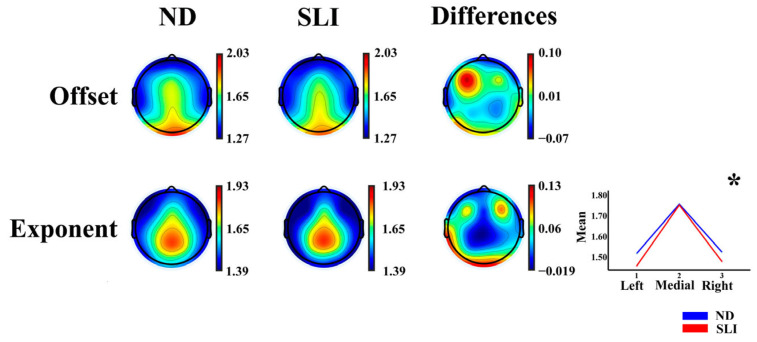
Topographies of the offset and exponent parameters for each group (normo-development (ND) and specific language impairment (SLI)) and the differences between the groups (ND−SLI). The asterisk indicates the interaction of the effects of laterality × group in the RM-ANOVA (marginal means are represented in the inset in the lower-right corner for this significant interaction).

Figure 2 shows the topographies of the offset and exponent parameters of the AP component and the differences between the groups (ND-SLI). The offset parameter displays a predominant distribution in the frontocentral and posterior areas, while the exponent is mainly distributed in the frontocentral and parietal areas.

The results of the RM-ANOVA analysis for the offset and exponent parameters of the AP component show a laterality × group interaction for the exponent parameter (F(1.81,108.498) = 3.48, *p* = 0.039, np2 = 0.055, power = 0.610). The post hoc results (Supplementary Table S4) did not survive the multiple comparison correction (FDR). Some laterality effects are reported in the description of Supplementary Table S5 considering its possible relationship with language lateralization.

**Figure 3:**
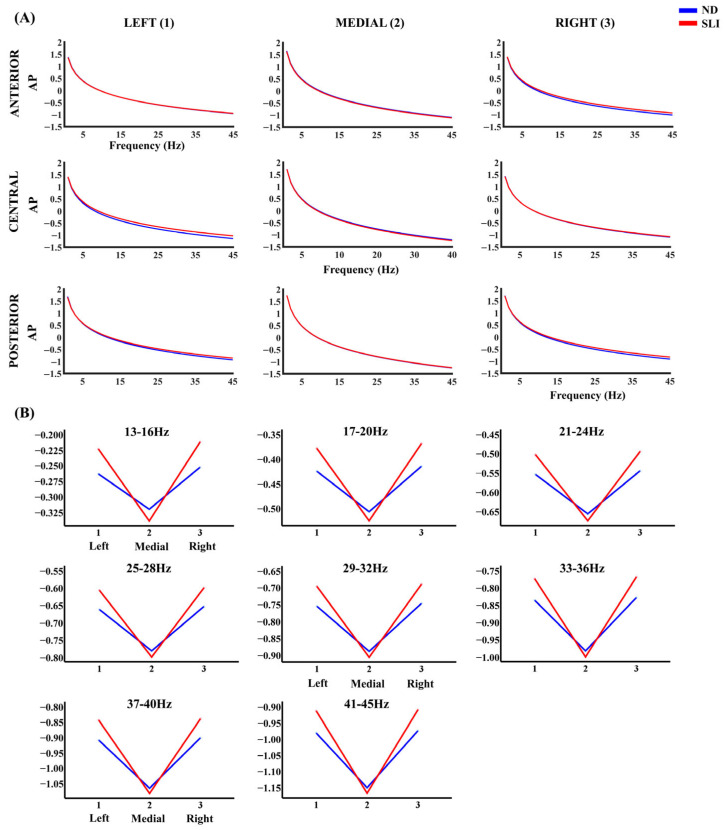
(**A**) Aperiodic component (AP) of the PSD across all frequencies and in each considered area for the ND group (blue line) and the SLI group (red line). (**B**) The marginal means of the AP PSD of the frequencies that showed significant effects of the interaction laterality × group in the RM-ANOVA.

Figure 3 shows the AP component of the PSD in each considered area. Table 2 presents the results of the RM-ANOVA analyzing the group effect for each frequency band of the AP component. Table 3 displays the RM-ANOVA exploring the group effect in the P component. Finally, Table 4 summarizes the significant effects of the post hoc analyses resulting from both analyses after multiple comparison correction (FDR), specifically highlighting the significant laterality × group or anterior–posterior x group interactions, which are described in the following paragraphs.

For the AP component, post hoc analyses show that, for the 13–16 Hz range, after FDR correction, the left–medial area difference (t(64) = 2.44, *p* = 0.047, d = 0.60) is higher for the SLI group (M = 0.114, SD = 0.099) compared to the ND group (M = 0.059, SD = 0.084). In addition, the right–medial area difference (t(64) = 2.19, *p* = 0.047, d = 0.054) shows higher values in the SLI group (M = 0.124, SD = 0.098) than the ND group (M = 0.069, SD = 0.105). For the 17–20 Hz range, interactions between group and laterality were also found. The left–medial area difference (t(64) = 2.53, *p* = 0.037, d = 0.62) is higher in the SLI group (M = 0.145, SD = 0.105) compared to the ND group (M = 0.085, SD = 0.088). Moreover, the right–medial area difference (t(64) = 2.30, *p* = 0.037, d = 0.06) is greater in the SLI group (M = 0.153, SD = 0.103) compared to the ND group (M = 0.094, SD = 0.107). Similar patterns are observed at higher frequencies. For 21–24 Hz, the left–medial area difference (t(64) = 2.58, *p* = 0.031, d = 0.63) is higher in the SLI group (M = 0.169, SD = 0.111) vs. the ND group (M = 0.105, SD = 0.093), as well as the right–medial area difference (t(64) = 2.37, *p* = 0.031, d = 0.058; SLI: M = 0.177, SD = 0.107; ND: M = 0.113, SD = 0.109). For 25–28 Hz, the left–medial area difference (t(64) = 2.61, *p* = 0.031, d = 0.638) is higher in the SLI group (M = 0.190, SD = 0.115) compared to the ND group (M = 0.122, SD = 0.097), as well as the right–medial area difference (t(64) = 2.41, *p* = 0.031, d = 0.060; SLI: M = 0.196, SD = 0.111; ND: M = 0.130, SD = 0.112). For 29–32 Hz, significant laterality × group interactions were observed. The difference between the left and medial areas (t(64) = 2.62, *p* = 0.026, d = 0.643) shows higher values in the SLI group (M = 0.207, SD = 0.119) compared to the ND group (M = 0.137, SD = 0.100), similarly to the difference between the right and medial areas (t(64) = 2.45, *p* = 0.026, d = 0.061; SLI: M = 0.213, SD = 0.114; ND: M = 0.144, SD = 0.114). For 33–36 Hz, laterality × group interactions were found regarding the left–medial area difference (t(64) = 2.63, *p* = 0.024, d = 0.646) and right–medial area difference; (t(64) = 2.48, *p* = 0.024, d = 0.061). For both, the SLI group had consistently higher scores (left vs. medial: M = 0.223, SD = 0.124; right vs. medial: M = 0.227, SD = 0.117) compared to the ND group (left vs. medial: M = 0.149, SD = 0.104; right vs. medial: M = 0.156, SD = 0.116). For 37–40 Hz, interactions were also observed in the left–medial area difference (t(64) = 2.64, *p* = 0.023, d = 0.648) and the right–medial area difference (t(64) = 2.50, *p* = 0.023, d = 0.062), being higher in the SLI group (left–medial: M = 0.237, SD = 0.127; right–medial: SLI: M = 0.240, SD = 0.119) compared to the ND group (left–medial: M = 0.161, SD = 0.107; right vs. medial: M = 0.167, SD = 0.118). Finally, significant interactions of Laterality × group were found for 41–45 Hz. The left–medial area difference (t(64) = 2.65, *p* = 0.022, d = 0.649) is higher in the SLI group (M = 0.251, SD = 0.131) compared to the ND group (M = 0.172, SD = 0.110), and the right–medial area difference (t(64) = 2.51, *p* = 0.022, d = 0.062) is higher in the SLI group (M = 0.255, SD = 0.122) compared to the ND group (M = 0.178, SD = 0.120). Supplementary Table S6 shows the significant interactions without the group effect.

#### 3.3.2. Periodic Component (P)

**Figure 4:**
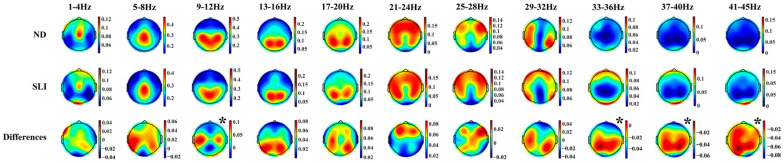
Topographies of the periodic component in the 11 collapsed frequency bands (4 Hz) for each group (ND and SLI) and the differences between them. The asterisk indicates significant differences between groups in the RM-ANOVA.

Figure 4 shows the topographies of the periodic component in both groups (ND and SLI) and their differences (ND-SLI). A well-defined regional distribution is observed for the different frequencies, with a higher intensity in the SLI group in high frequencies.

**Figure 5:**
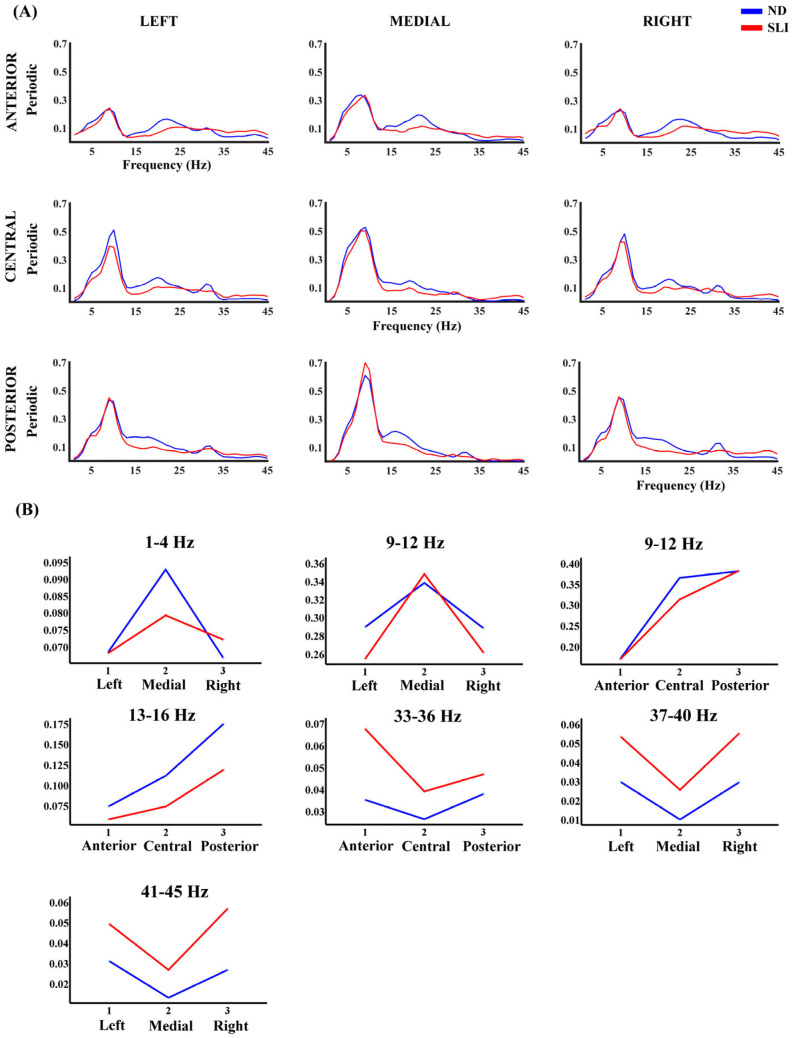
(**A**) Periodic component of the PSD across all frequencies and in each considered area for the ND group (blue line) and SLI group (red line). (**B**) Marginal means of the significant interaction effects of the factors Laterality × group and antero-posterior x group in the RM-ANOVA.

Figure 5 shows the periodic component’s power values (P) in each defined area. The RM-ANOVA results for the P component of the PSD revealed significant group differences across specific frequency ranges (Table 3). Post hoc analyses (summary in Table 4) indicated that, in the 37–40 Hz range (t(64) = 3.27, *p* = 0.003, d = 0.787), the SLI group (M = 0.047, SD = 0.039) had significantly higher values compared to the ND group (M = 0.022, SD = 0.020). Similarly, in the 41–45 Hz range (t(64) = 3.23, *p* = 0.004, d = 0.774), the SLI group (M = 0.048, SD = 0.040) had higher values than the ND group (M = 0.023, SD = 0.019). Interactions of the effect of the group with laterality were also found. Additionally, for the same frequency range (9–12 Hz), a significant antero-posterior x group interaction was found. In this case, the difference between the central and anterior areas (t(64) = 2.29, *p* = 0.038, d = 0.057) is higher in the ND group (M = 0.194, SD = 0.101) compared to the SLI group (M = 0.142, SD = 0.076). Moreover, the difference between the posterior and central areas (t(64) = 2.37, *p* = 0.038, d = 0.058) shows higher values in the SLI group (M = 0.066, SD = 0.078) than in the ND group (M = 0.017, SD = 0.086). In the 33–36 Hz range, an antero-posterior × group interaction was found. Post hoc analyses revealed that the anterior area (t(64) = 2.97, *p* = 0.013, d = 0.72) had significantly higher values in the SLI group (M = 0.069, SD = 0.058) compared to the ND group (M = 0.035, SD = 0.034). For the 41–45 Hz range, a significant Laterality × group interaction was observed, with differences across all three areas. In the left area (t(64) = 2.46, *p* = 0.035, d = 0.591), the SLI group (M = 0.054, SD = 0.048) showed significantly higher values compared to the ND group (M = 0.031, SD = 0.026). A similar pattern was found in the medial area (t(64) = 2.29, *p* = 0.035, d = 0.551), where the SLI group (M = 0.029, SD = 0.038) had higher values than the ND group (M = 0.013, SD = 0.020). Finally, the right area (t(64) = 3.86, *p* = 0.002, d = 0.927) exhibited the most pronounced difference, with the SLI group (M = 0.060, SD = 0.047) showing significantly higher values than the ND group (M = 0.026, SD = 0.024). In Supplementary Table S7, we show the significant interactions without the group effect. Significant post hoc results that did not survive multiple-comparisons correction are shown in Supplementary Table S8.

As a complementary robustness check, an additional RM-ANOVA analysis was performed after correcting for possible outliers (z-score > 3) in the periodic and aperiodic components. This analysis revealed a slight improvement in the stability and statistical robustness of the previously reported effects, as well as some additional significant interactions (see Supplementary Table S9). Notably, the proportion of corrected data remained low across all groups and components (ND: aperiodic = 0.79%, periodic = 1.77%; SLI: aperiodic = 0.07%, periodic = 1.25%).

## 4. Discussion

This study analyzed brain activity at rest in a group of children diagnosed with SLI compared to an ND group. The objective was, on one hand, to assess the stability and adaptability of neuronal activity measured through MSE. On the other hand, the study aimed to examine more precisely the patterns of neuronal activity underlying this disorder, measured with an innovative approach that decomposes the PSD into its AP and P components.

### 4.1. Multiscale Entropy (MSE)

Our findings show an increase in MSE values across the scales in both groups, but with a non-significant tendency toward lower values in the SLI group. These findings are consistent with previous studies on complexity in neurodevelopmental disorders, such as ADHD [61,62,63,64] and ASD [65,66,67,68], which have reported lower variability in neural network dynamics. However, the absence of significant differences between the groups suggests that the variability in the neural dynamics of children with SLI would be more similar to that of typically developing children, indicating that their adaptability, although slightly reduced, is not as compromised as in other neurodevelopmental disorders.

### 4.2. Parametrization of Fitting Oscillations and One over F (FOOOF)

#### 4.2.1. Topographies of the Exponent and Offset Parameters of the Aperiodic Component

The topographies of the AP component parameters (offset and exponent) show independence in the regional distributions for each parameter, consistent with previous studies [110,111]. The offset parameter presents a predominant distribution in the frontocentral and posterior regions, with higher values in posterior areas, and the exponent parameter is primarily distributed in frontocentral and parietal areas, being consistent with previous results [106]. These findings suggest that the offset and exponent reflect distinct neurophysiological mechanisms. While the offset is related to the overall cortical excitability and global electroencephalogram amplitude [112,113,114], its distribution suggests that it may be influenced by the default mode network [115,116]. On the other hand, the exponent is linked to the spectral organization of neuronal activity and the balance between excitatory and inhibitory processes [79,80], and it seems to focus on regions crucial for cognitive control and sensory integration. The lower value of the exponent in the SLI group could indicate alterations in cortical dynamics, with the impaired maturation of the balance of excitatory and inhibitory synaptic currents (E/I, balance), as observed in other neurodevelopmental disorders [81,83,84,85], suggesting alterations in neuronal communication efficiency and sensory and cognitive processing in children with SLI. This change in the E/I balance, indexed by the decrease in the exponent in the SLI group, could have been due to a reduced inhibitory or some increased excitatory synaptic currents in the SLI group during development.

#### 4.2.2. Aperiodic Component (AP)

The analysis of the aperiodic component (AP) shows significant interactions between the laterality and group, with higher aperiodic values in the SLI group in the 13–45 Hz frequency range. These alterations in the AP component suggest the atypical modulation of background activity and increased cortical excitability in the lateral areas in the SLI group. The elevated aperiodic component values in this same region for the SLI group, if due to a lower exponent—as suggested by the statistical trend for a lower exponent in SLI—would suggest an atypical E/I balance with a bias for excitatory activity [79,80].

These spectral differences in lateralized regions could be related to dysfunctions in neural connectivity, which have been associated in the literature with language development [117,118]. However, as this study did not include direct measures of functional connectivity, this interpretation should be viewed with caution and considered as a hypothesis for future studies integrating measures of connectivity and language performance.

#### 4.2.3. Topographies of the Periodic Component

Moreover, the topographies of the P component exhibit a well-defined regional distribution across the different frequency ranges analyzed. This regional organization aligns with the canonical frequency bands described in the literature, including delta (1–4 Hz), theta (4–8 Hz), alpha (8–12 Hz), beta (12–30 Hz) and gamma (>30 Hz) [49,50,77,119,120,121]. The low P beta topography (13–20 Hz) is slightly different from the canonical PSD beta topography given that the posterior frequency in P beta is prolonged to higher frequencies than in the canonical beta. The distribution patterns observed in this study are consistent with those reported in a previous parametrization analysis conducted on a sample of 240 neurotypical participants [106]. The increased intensity at higher frequencies (33–45 Hz) in the SLI group suggests that, while the spectral organization of low-frequency (1–8 Hz) oscillations remains preserved in these children, differences emerge in the magnitude of this organization at higher frequencies. This finding may indicate that, although the fundamental architecture of rhythmic neural activity is not disrupted in SLI, the deficits observed in language processing and other cognitive functions, such as attention [40,122], memory [123] and executive function [124,125], could be associated with reduced neuronal synchronization efficiency, impairing the modulation of high-frequency oscillatory activity in these children.

#### 4.2.4. Periodic Component (P)

The results of the group comparison for the P component show an increase in power at high frequencies (37–45 Hz) in the SLI group compared to the ND group. Previous studies have reported an increase in activity in slower bands (delta and theta) and a decrease in faster bands (alpha and beta) in children with SLI [73,74,75,76]. However, our results indicate a different pattern in the high-frequency gamma band (33–45 Hz), which has been linked to processes of sensory integration, neuronal synchronization and cognitive modulation [126]. An increase in the gamma P band in SLI could reflect an imbalance, once again, in the regulation of cortical excitability, possibly related to the reduced efficiency of inhibitory mechanisms, as suggested by the AP PSD component, increasing the neuronal capacity for the generation of synchronization for high frequencies [126] in children diagnosed with SLI.

These results are not homogeneous across all regions or high frequencies but instead vary based on the laterality (41–45 Hz) and antero-posterior distribution (33–36 Hz). Thus, we found (i) greater high-frequency power (41–45 Hz) in the SLI group in the left, medial and right areas, suggesting a disruption in the synchronization of interhemispheric networks, and (ii) greater power in the anterior area for the SLI group (33–36 Hz), suggesting an impairment in intra-hemispheric synchronization. This could be related to alterations in executive control and attentional modulation—functions that are strongly associated with activity in the prefrontal cortex [127]. These findings, on the one hand, reinforce previous evidence of structural alterations in lateralized regions (left and right) in children with SLI [22,27]. Additionally, they suggest that these differences are not solely explained by an increase or decrease in certain frequency bands but by an atypical pattern of oscillatory modulation between regions, which could impact the intra- and inter-hemispheric functional dynamics of the SLI brain.

On the other hand, the results for the 9–12 Hz range, corresponding to the canonical alpha band [128], show that the difference between the central area and the anterior area is greater in ND children, which is similar to the results of Stanojević et al. [76], while the difference between the posterior area and the central area is greater in children with an SLI diagnosis. This suggests an antero-posterior alteration of these frequencies and the modulation of oscillatory activity. Previous studies have indicated that alpha rhythm modulation is key for corticocortical and intracortical coordination [129], as well as for the integration of sensory and linguistic information [130,131]. The differences found in this study, regarding the distribution of alpha oscillatory activity, could be related to difficulties in neuronal synchronization between regions associated with processes such as cortical inhibition, attention regulation and language [130], as well as with the modulation of sensory perception [131] in children with SLI.

### 4.3. Limitations

While these findings highlight the importance of research on how brain activity in neurodevelopmental disorders such as SLI could reflect alterations in the underlying neuronal dynamics, this study presents some limitations that must be considered. Firstly, the small sample size could have led to the lack of significance in some of our results, such as the MSE, although the observed trend suggests similarities with other disorders, such as ADHD [64] and ASD [68]. Moreover, the different tests used for the delimitation of the diagnostic threshold do not permit the computation of correlations between behavioral tests and EEG-derived parameters. A methodological limitation of this study is that no correction for multiple comparisons was applied to the *p*-values obtained in the RM-ANOVA models by frequency band. Although FDR correction was applied to the post hoc analyses, the band omnibus models were not corrected. This decision was based on the fact that the main objective was to identify general patterns of interaction and differences between groups, rather than to derive independent statistical inferences for each frequency band. However, we recognize that this approach may increase the risk of type I errors, so the results should be interpreted with caution. Furthermore, although our main analyses were conducted without modifying the outliers, a complementary robustness analysis using z-score correction revealed consistent and even strengthened effects in some cases. These results support the robustness of our findings despite the presence of a small number of outliers. However, we recognize that the definition of outliers in physiological data is context-dependent, and future studies could further explore this aspect using robust statistical methods. Despite these limitations, the findings of this study provide a better understanding of the differences in connectivity, measured indirectly, and the modulation of brain activity, which could have implications for diagnosis and the development of interventions to improve cognitive and linguistic performance in these children.

## 5. Conclusions

This study analyzed brain activity in children with specific language impairment (SLI) compared to typically developing children (ND), using measures of neural complexity (MSE) and the decomposition of the power spectrum (PSD) into its periodic (P) and aperiodic (AP) components. Although no significant differences were observed in the multiscale entropy (MSE) or in the overall topographical organization of the AP and P components, statistically significant differences were identified in specific frequency bands and specific brain regions, after correction for multiple comparisons. These alterations could reflect changes in spectral dynamics linked to neurophysiological processes such as cortical excitation–inhibition balance or neuronal synchronization, particularly at high frequencies and in the alpha band. It is important to note that no direct functional connectivity analyses were performed, nor was the involvement of networks such as the default mode network (DMN) assessed, so such interpretations should be viewed with caution.

From a clinical perspective, these findings underline the value of analyzing the AP and P components of the PSD separately to identify atypical neuroelectrical patterns in children with language difficulties. In the future, the more precise characterization of these differences could contribute to the development of EEG biomarkers that support early diagnosis and the personalization of interventions in SLI. However, further studies should include direct measures of functional connectivity and correlations with linguistic and cognitive performance to confirm and extend these results.

## Figures and Tables

**Table 1:** Means (M) and standard deviations of the specific language impairment (SLI) and normo-developmental (ND) groups.

	SLI	ND
Age	M = 6.38, SD = 1.75	M = 6.90, SD = 2.08
Males	M = 6.52, SD = 1.71	M = 7.05, SD = 2.09
Females	M = 6.09, SD = 1.87	M = 6.29, SD = 2.05
KBIT	M = 22.52, SD = 7.49	M = 26.63, SD = 6.49
Components	M = 12.93, SD = 0.254	M = 12.56, SD = 0.652
Epochs	M = 27.87, SD = 2.45	M = 32.58, SD = 7.51

**Table 2:** Significant group interactions were obtained in the RM-ANOVA of the aperiodic component (AP) of the power spectral density (PSD) with the following factors: subject group (ND and SLI), antero-posterior distribution (anterior, central, posterior) and laterality (left, medial, right). Each RM-ANOVA was performed for each frequency collapse (4 Hz, total 11 frequency ranges). Age in days and biological sex were used as covariates.

Frequency	Within Subjects
13–16 Hz	Laterality × group *p* = 0.034 F(1.89,114.96) = 3.58, np2 = 0.055, power = 0.636
17–20 Hz	Laterality × group *p* = 0.025 F(1.88,114.93) = 4.52, np2 = 0.060, power = 0.676
21–24 Hz	Laterality × group *p* = 0.021 F(1.89,115.11) = 4.12, np2 = 0.063, power = 0.701
25–28 Hz	Laterality × group *p* = 0.018 F(1.89,115.36) = 4.27, np2 = 0.065, power = 0.719
29–32 Hz	Laterality × group *p* = 0.016 F(1.89,115.63) = 4.38, np2 = 0.067, power = 0.731
33–36 Hz	Laterality × group *p* = 0.015 F(1.90,115.90) = 4.47, np2 = 0.068, power = 0.741
37–40 Hz	Laterality × group *p* = 0.014 F(1.90,116.16) = 4.54, np2 = 0.069, power = 0.748
41–45 Hz	Laterality × group *p* = 0.013 F(1.91,116.43) = 4.60, np2 = 0.070, power = 0.755

**Table 3:** Significant group interactions obtained in the RM-ANOVA of the periodic component (P) of the power spectral density (PSD) with the following factors: subject group (ND and SLI), antero-posterior distribution (anterior, central, posterior) and laterality (left, medial, right). Each RM-ANOVA was performed for each frequency collapse (4 Hz, total 11 frequency ranges). Age in days and biological sex were used as covariates.

	RM-ANOVA	
Frequency	Within Subjects	Between Subjects
1–4 Hz	Laterality × group *p* = 0.050 F(1.98,120.93) = 3.07, np2 = 0.048, power = 0.581	-
9–12 Hz	Laterality × group *p* = 0.017 F(1.57,95.48) = 4.78, np2 = 0.073, power = 0.710 Antero-posterior × group *p* = 0.031 F(1.97,120.13) = 3.59, np2 = 0.056, power = 0.650	-
13–16 Hz	Antero-posterior × group *p* = 0.030 F(1.89,115.23) = 3.69, np2 = 0.057, power = 0.650	-
33–36 Hz	Antero-posterior × group *p* = 0.036 F(1.53,93.23) = 3.84, np2 = 0.059, power = 0.603	-
37–40 Hz	-	Group *p* = 0.005 F(1,61) = 8.61, np2 = 0.124, power = 0.823
41–45 Hz	Laterality × group *p* = 0.037 F(1.97,120.09) = 3.40, np2 = 0.053, power = 0.626	Group *p* = 0.006 F(1,61) = 8.03, np2 = 0.116, power = 0.796

**Table 4:** Summary of post hoc analysis for aperiodic and periodic components across selected frequency ranges (4 Hz). Only significant group effect remains after false discovery rate (FDR) correction.

Aperiodic		
Frequency Range	Between Subjects	Within Subjects
13–16 Hz	-	Left–Medial (SLI > ND) Right–Medial (SLI > ND)
17–20 Hz	-	Left–Medial (SLI > ND) Right–Medial (SLI > ND)
21–24 Hz		Left–Medial (SLI > ND) Right–Medial (SLI > ND)
25–28 Hz	-	Left–Medial (SLI > ND) Right–Medial (SLI > ND)
29–32 Hz	-	Left–Medial (SLI > ND) Right–Medial (SLI > ND)
33–36 Hz	-	Left–Medial (SLI > ND) Right–Medial (SLI > ND))
37–40 Hz	-	Left–Medial (SLI > ND) Right–Medial (SLI > ND)
41–45 Hz	-	Left–Medial (SLI > ND) Right–Medial (SLI > ND)
**Periodic**		
9–12 Hz	-	Central–Anterior (ND>SLI) Posterior–Central (SLI > ND)
33–36 Hz	-	Anterior (SLI > ND)
37–40 Hz	SLI > ND	-
41–45 Hz	SLI > ND	Left (SLI > ND) Medial (SLI > ND) Right (SLI > ND)

## Data Availability

The data and code related to the present study are available under reasonable request to the corresponding author (bangulo@us.es).

## Supplementary Materials

The following supporting information can be downloaded at: https://www.mdpi.com/article/10.3390/e27060572/s1. Supplementary Figure S1: Goodness-of-fit histograms; Supplementary Table S1: Description of the types of MSE scales; Supplementary Table S2: Areas of interest; Supplementary Table S3: RM-ANOVA of the MSE; Supplementary Table S4: Post-hoc analysis of exponent parameter; Supplementary Table S5: RM-ANOVA of exponent and offset parameters; Supplementary Table S6: RM-ANOVA of aperiodic component; Supplementary Table S7: RM-ANOVA of periodic component; Supplementary Table S8: Post-hoc analysis of the periodic component; Supplementary Table S9: Additional significant effects observed in the RM-ANOVA of Aperiodic and Periodic components

## Abbreviations

The following abbreviations are used in this manuscript:

ADHD	Attention-Deficit Hyperactivity Disorder
AP	Aperiodic
ASD	Autism Spectrum Disorder
ASR	Artifact Subspace Reconstruction
CELF	Clinical Evaluation of Language Fundamentals
DTI	Diffusion Tensor Imaging
EEG	Electroencephalogram
ERPs	Event-Related Potentials
FDR	False Discovery Rate
fMRI	Functional Magnetic Resonance Imaging
FOOOF	Fitting Oscillations and One Over F
fTCD	Functional Transcranial Doppler
ICA	Independent Component Analysis
ITPA	Illinois Test of Psycholinguistic Abilities
KBIT	Kaufman Brief Intelligence Test
M	Mean
MAE	Mean Absolute Error
MMN	Mismatch Negativity
MSE	Multiscale Entropy
ND	Normo-Development
P	Periodic
PLON-R	Navarre Oral Language Test—Revised
PSD	Power Spectral Density
PPVT-5	Peabody Picture Vocabulary Test
RM-ANOVA	Repeated-Measures Analysis of Variance
SD	Standard Deviation
SE	Sample Entropy
SLI	Specific Language Impairment
SPECT	Single-Photon Emission Computed Tomography
UDIATE	Unidad de Desarrollo Infantil y Atención Temprana
