# Supplementary material for: Brain Complexity and Parametrization of Power Spectral Density in Children with Specific Language Impairment
Source: Entropy (Basel). 2025 May 28;27(6):572. doi: 10.3390/e27060572 (PMC12191662; doi:10.3390/e27060572)
Supplement: Supplementary file 1 [file entropy-27-00572-s001.zip › entropy-3594963-supplementary.pdf]

SUPPLEMENTARY MATERIAL

Supplementary Table S1

Detailed description of the number of points, sample periods, and frequencies covered in three types of scales in MSE: fine, medium, and coarse.

Types scales	Scales	Sampling period (ms)	Sampling frequency (Hz)	number points trial	Higher Frequency (Nyquist Hz)	Frequency Resolution (Hz)
Fine	1	0.9766	1024	4096	512	0.25
	**25**	**24.41**	**40.96**	**163**	**20.48**	**0.25**
Medium	26	25.39	39.38	157	19.69	0.25
	**46**	**44.92**	**22.26**	**89**	**11.13**	**0.25**
Coarse	47	45.89	21.78	87	10.89	0.25
	136	132.8125	7.529	30	3.764	0.25

Supplementary Table S2

Description of collapsed electrodes to define areas of interest

Area	Electrodes
Left-Anterior	Fp1, F3, F7
Left-Central	C3, T3
Left-Posterior	P3, O1, T5
Medial-Anterior	Fz
Medial-Central	Cz
Medial-Posterior	Pz
Right-Anterior	Fp2, F4, F8
Right-Central	C4, T4
Right-Posterior	P4, O2, T6

Supplementary Table S3

Significant results obtained in the RM-ANOVA of MSE with factors: group of subjects (Normal development (ND) and Specific Language Impairment (SLI)), scales (fine, medium, and coarse), antero-posterior (anterior, central, posterior), and laterality (left, medial, right), and age in days and gender as covariates.

**Within-Subjects**	
Scales p=<.001	F(1.62,98.96)=79.57, np2=.566, power=1
Scales x laterality p<.001	F(2.52,153.40)=8.01, np2=.116, power=.978

Note: The post hoc analysis of the interaction between scales x laterality shows in the fine scale, higher values of the left area in the difference between left area – medial area (t(65) = 18.39, p < .001) and in the difference between left area – right area (t(65) = 2.67, p = .010), while the right area is higher in the difference between right area – medial area (t(65) = 17.37, p < .001). In the medium scale, the medial area presents a higher value in the difference between medial area – left area (t(65) = 3.79, p < .001) and in the difference between medial area – right area (t(65) = 4.57, p < .001). Finally, on the coarse scale, the medial area shows a higher value in the difference between the medial area – left area (t(65) = 12.84, p < .001) and in the difference between the medial area – right area (t(66) = 12.91, p < .001).

Supplementary Table S4

Results of the post-hoc analysis of exponent parameter of the aperiodic component of Power Spectral Density (PSD) in the groups with Specific Language Impairment (SLI) and with normo-development (ND), which were significant before correction for multiple comparisons, but did not remain after applying said correction.

Areas	t-test	Mean (M) AND Standard Deviation (SD)
medial - left area	t(64) = 2.03, p = .047, d = .51	SLI (M = .289, SD = .098)<br>ND (M = .239, SD = .099)
medial - right area	t(64) = 2.02, p = .047, d = .50	SLI (M = .273, SD = .080)<br>ND (M = .231, SD = .087)

Supplementary Table S5

Significant results obtained in the RM-ANOVA of exponent and offset of the aperiodic component of Power Spectral Density (PSD) with factors: group of subjects (ND and SLI), antero-posterior (anterior, central, posterior) and laterality (left, medial, right). Age in days and gender as covariates. Asterisk shows interactions with the group.

Within-Subjects	
**Offset**	**Exponent**
Laterality p<.001<br>F(1.74,104.28) = 8.96, np2 = .130, power = .953	Laterality p<.001<br>F(1.81,109.49) = 15.53, np2 = .206, power = .999
Antero-posterior p<.001<br>F(1.58,94.03) = 9.92, np2 = .142, power = .957	Antero-posterior p = .002<br>F(1.59,95.08) = 7.78, np2 = .115, power = .903

Note: Post hoc laterality analysis of the offset parameter shows a higher value in the medial area for the difference between medial area and left area (t(65) = -16.39, p < .001) and for the difference between medial area and right area (t(65) = 13.93, p < .001). Furthermore, the right area shows a higher value for the difference between right area and left area (t(65) = 2.44, p = .017). Post hoc laterality analysis of the exponential parameter shows a higher value in the medial area for the difference between medial area and left area (t(65) = 20.94, p < .001) and for the difference between left medial area and right area (t(65) = 23.62, p < .001).

Supplementary Table S6

Significant results obtained in the RM-ANOVA of aperiodic components of the power spectral density (PSD) with the factors: subject group (controls and SLI), antero-posterior (anterior, central, posterior) and laterality (left, medial, right). Each RM-ANOVA was performed for each frequency collapse (4Hz, total 11 frequency ranges). Age in days and gender were used as covariates.

Frequency	Within-subjects
**1-4Hz**	Antero-posterior p<.001<br>F(1.58,98.84)=10.21, np2=.143, power=.963
**5-8Hz**	Antero-posterior p<.001<br>F(1.66,100.95)=10.60, np2=.148, power=.974
**9-12Hz**	Laterality p=.041<br>F(1.89,115.48)=3.37, np2=.052, power=.608<br>Antero-posterior p<.001<br>F(1.76,107.17)=9.52, np2=.135, power=.962
**13-16Hz**	Laterality p=.015<br>F(1.89,114.96)=4.45, np2=.068, power=.736<br>Antero-posterior p=.001<br>F(1.88,112.08)=8.38, np2=.121, power=.949
**17-20Hz**	Laterality p=.007<br>F(1.88,114.93)=5.31, np2=.080, power=.813<br>Antero-posterior p=.001

**21-24Hz**	F(1.89,115.43)=7.40, np2=.108, power=.926<br>Laterality p=.004<br>F(1.89,115.11)=5.99, np2=.090, power=.860<br>Antero-posterior p=.002<br>F(1.93,122)=6.60, np2=.098, power=.897
**25-28Hz**	Laterality p=.002<br>F(1.89,115.36)=6.56, np2=.097, power=.891<br>Antero-posterior p=.004<br>F(1.95,118.86)=5.94, np2=.089, power=.865
**29-32Hz**	Laterality p=.002<br>F(1.89,115.63)=7.02, np2=.103, power=.912<br>Antero-posterior p=.006<br>F(1.96,119.59)=5.39, np2=.081, power=.830
**33-36Hz**	Laterality p=.001<br>F(1.90,115.90)=7.41 np2=.108, power=.926<br>Antero-posterior p=.009<br>F(1.97,119.94)=4.93, np2=.075, power=.794<br>Laterality x antero-posterior p=.052<br>F(3.77,231.46)=2.43, np2=.038, power=.674
**37-40Hz**	Laterality p=.001<br>F(1.90,116.16)=7.74, np2=.113, power=.937<br>Antero-posterior p=.013<br>F(1.97,120.04)=4.54, np2=.069, power=.759<br>Laterality x antero-posterior p=.049<br>F(3.78,231)=2.47, np2=.039, power=.682
**41-45Hz**	Laterality p=.001<br>F(1.91,116.43)=8.06, np2=.117, power=.947<br>Antero-posterior p=.018<br>F(1.97,119.97)=4.17, np2=.064, power=.720<br>Laterality x antero-posterior p=.046<br>F(3.78,230.58)=2.51, np2=.039, power=.689

Supplementary Table S7

Significant results obtained in the RM-ANOVA of periodic components (P) of the power spectral density (PSD) with the factors: subject group (ND and SLI), antero-posterior (anterior, central, posterior) and laterality (left, medial, right). Each RM-ANOVA was performed for each frequency collapse (4Hz, total 11 frequency ranges). Age in days and gender were used as covariates.

Frequency	Within-subjects
**5-8Hz**	Laterality p<.001<br>F(1.29,79.27)=15.08, np2=.198, power=.989
**9-12Hz**	Laterality p=.026<br>F(1.57,95.48)=4.19, np2=.064, power=.65<br>Antero-posterior p=.017<br>F(1.97,120.128)=4.25, np2=.065, power=.729<br>Laterality x antero-posterior p=.024<br>F(3.33,203.06)=3.08, np2=.048, power=.747
**21-24Hz**	Laterality p=.041<br>F(1.84,112.112)=3.38, np2=.053, power=.602<br>Laterality x antero-posterior p=.046<br>F(2.94, 179.46)=2.74, np2=.043, power=.651
**25-28Hz**	Laterality p=.025<br>F(1.73,107.47)=4.06, np2=.062, power=.666<br>Antero-posterior p=.023<br>F(1.68,102.22)=4.25, np2=.065, power=.678
**37-40Hz**	Antero-posterior p=.014<br>F(1.54,93.81)=5.07, np2=.077, power=.731

**41-45Hz**	Antero-posterior p=.015 F(1.78,108.48)=4.64, np2=.071, power=.737

Supplementary Table S8

Results of the post-hoc analysis of the periodic component of the power spectral density (PSD) in the groups with Specific Language Impairment (SLI) and with normo-development (ND), which were significant before correction for multiple comparisons, but did not remain after applying said correction.

Frequency	Areas	t-test	Mean (M) AND Standard Deviation (SD)
1 - 4Hz	right - medial area	t(64) = 2.11, p = .038, d = .52	SLI (M = .009, SD = .034)<br>ND (M = .025, SD = .031)
9 - 12Hz	medial - left area	t(64) = 2.44, p = .018, d = .59	SLI (M = .091, SD = .074)<br>ND (M = .050, SD = .063)
9 – 12Hz	Medial – right area	t(64) = 2.06, p = .043, d = .51	SLI (M = .085, SD = .073)<br>ND = (M = .050, SD = .062)
13 – 16Hz	posterior	t(64) = 2.23, p = .030, d = .549	SLI (M = .113, SD = .117)<br>ND ((M = .176 SD = .112)

Supplementary Table S9

Additional significant effects observed in the RM-ANOVA of aperiodic (AP) and periodic (P) components analyses after z-score-based outlier correction

Frequency	AP	P
**5-8Hz**	Within-subjects:<br>Laterality x group p=.038<br>F(1.95,118.66)=3.39, np2=.053, power=.621	
**9-12Hz**	Laterality x group p=.019<br>F(1.93,117.92)=4.16, np2=.064, power=.714	
**13-16Hz**		Between-subjects p=.005<br>F(1,61)=8.54, np2=.123, power=.820
**17-20Hz**		Between-subjects p=.005<br>F(1,61)=8.32, np2=.120, power=.810
**33-36Hz**		Within-subjects:<br>Laterality x group p=.046<br>F(1.95,118.76)=3.20, np2=.050, power=.594<br>Between-subjects p=.015<br>F(1,61)=6.22, np2=.093, power=.690

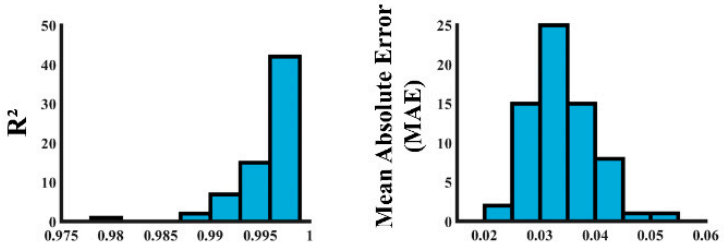

Supplementary Figure S1. Histograms of explained variance (R^2) and mean absolute error (MAE) metrics to assess the goodness-of-fit measures between model and canonical power spectrum de ND y SLI.
